# Unrolling the Mat: A Mixed-Methods Study of Yoga Awareness, Perceptions, and Barriers to Daily Practice Among MBBS Students

**DOI:** 10.7759/cureus.87306

**Published:** 2025-07-04

**Authors:** Sachin B Rathod, Smita R Sorte, Mrunal Phatak, Atul K Sharma, Kratika Mulchandani

**Affiliations:** 1 Physiology, All India Institute of Medical Sciences, Nagpur, Nagpur, IND

**Keywords:** holistic lifestyle interventions, lifestyle disorders, mbbs students’ perceptions, stress management in medical students, student well-being, undergraduate health education, yoga adherence barriers, yoga in medical education, yoga integration strategies, yoga practice readiness

## Abstract

Introduction

Yoga, rooted in Indian tradition, is recognized for its holistic health benefits. With a growing global emphasis on preventive health, yoga has gained attention for its capacity to mitigate stress, improve concentration and enhance general well-being. Burdened with academic pressures, irregular routines and sedentary lifestyles, medical students represent a group that could significantly benefit from incorporating yoga into their daily lives. This study aims to bridge this gap by evaluating MBBS students’ awareness, perceptions, readiness to adopt yoga and the practical challenges they face, along with their views on integrating structured yoga sessions into their curriculum.

Methods

A cross-sectional study was conducted at AIIMS, Nagpur, among MBBS students, with a total of 155 students (100%) from the 2019 to 2023 batches. A structured, pre-validated questionnaire (Cronbach’s α=0.973) was administered electronically using a Google Form link shared via student WhatsApp groups. The survey comprised five sections: awareness about yoga, perceptions about its preventive role in lifestyle disorders, readiness to integrate it into daily life, barriers to practice and preferences regarding formal integration of yoga practice in the MBBS curriculum. Responses were recorded on 5-point Likert scales. Data analysis was performed using Python (Jupyter) for cleaning, statistics and reliability testing, and Jamovi (v2.3.28) for non-parametric tests due to non-normal distribution.

Results

The findings revealed a high awareness among participants, with an average awareness score (87.6%), high agreement on yoga’s role in stress reduction (4.45%) and overall health (4.63%). Perception of yoga as preventive and therapeutic for lifestyle disorders was strong (mean=84.0%). Batch-wise differences were noted in yoga awareness scores (p<0.001), and interns were more likely to endorse yoga as a complementary therapy (p=0.037). The readiness score was 68.2%, with only a 3.41/5 average agreement on willingness to integrate yoga daily. A significant gender-based difference was observed for perceived physical preparedness (p=0.035) and preference for educational content in yoga, with males scoring higher. Major barriers included time constraints (112, 72.2%), inconsistency (93, 60%) and lack of motivation to wake up early (84, 54.54%). Overall, 140 (90.3%) agreed that yoga practice should be part of the MBBS curriculum. Most preferred physical sessions (116, 74.84%), held three days a week (63, 40.65%), for 20-30 mins (93, 61.59%), with a focus on postures, breathing, meditation and stress relief. Some students preferred sessions to be optional and scheduled flexibly around academic hours.

Conclusion

This study highlights a significant gap between high levels of awareness and relatively low readiness to incorporate yoga into daily life among MBBS students. Despite recognizing its benefits, students face notable practical challenges, especially time management and consistency. However, the overwhelming support for integrating structured yoga sessions into the medical curriculum presents an actionable opportunity. Addressing identified barriers through flexible scheduling, optional participation and an emphasis on stress management may enhance adoption. These findings underscore the potential of yoga not just as a wellness tool but as an integral component of medical education aimed at fostering personal resilience and long-term professional well-being.

## Introduction

Yoga, an ancient Indian discipline, integrates physical postures (asanas), controlled breathing (pranayama), and meditation to foster physical, mental, and emotional well-being. In recent decades, yoga has evolved from a spiritual pursuit to a globally acknowledged health-promoting practice, endorsed by leading healthcare organizations for its role in managing stress, enhancing resilience, and preventing non-communicable diseases [1]. In India, yoga’s public health value has been institutionalized through the Ministry of Ayush and the nationwide observance of the International Day of Yoga [2].

Medical students, as future healthcare providers, face unique stressors including academic overload, long study hours, irregular schedules, and clinical responsibilities [3]. This environment often contributes to physical inactivity, poor sleep, and psychological strain. Studies have shown a high prevalence of anxiety, burnout, and lifestyle disorders among medical undergraduates, which can affect both personal health and professional development [4]. Yoga, by its calming and strengthening effects, is particularly well-suited for this population, offering a self-regulated, non-pharmacological strategy to manage stress and build long-term healthy habits.

Abdulghani et al. demonstrated yoga’s efficacy in a randomized study of Saudi medical students that a six-week yoga program significantly improved sleep quality (Pittsburgh Sleep Quality Index (PSQI): -2.3 ± 0.7, p < 0.001), reduced anxiety scores by 30% (Generalized Anxiety Disorder-7 scale (GAD-7): -4.6 ± 1.2, p < 0.001), and lengthened sustained-attention time by 18% compared with wait-list controls [5].

Despite these documented benefits, the adoption of yoga among MBBS students remains inconsistent. Factors such as lack of time, academic demands, absence of institutional support, and misconceptions about yoga contribute to low engagement. Sociocultural factors, such as traditional family attitudes, misconceptions that yoga conflicts with certain belief systems, and urban-rural disparities in access to qualified instructors, discourage some students from participating. Gender-specific issues also contribute: many female students feel uneasy about mixed-gender classes, exercise attire, while some male peers dismiss mind-body practices as insufficiently athletic. Finally, systemic limitations within medical campuses, including cramped hostel rooms, a lack of dedicated practice spaces, the absence of timetabled wellness periods, and inadequate institutional funding for qualified yoga trainers, collectively impede regular engagement. While the National Medical Commission (NMC) has mandated yoga sessions during orientation programs [6], and some institutions offer yoga as part of elective modules, the structured integration of daily yoga practice into the formal medical curriculum remains rare.

To provide a structured understanding of student engagement with yoga, this study is conceptually grounded in the Health Belief Model (HBM). The HBM posits that health-related behaviors are influenced by individuals’ perceptions of the severity of a health issue, their susceptibility to it, the perceived benefits of taking preventive action, and the perceived barriers to doing so. Applied in the context of this study, students’ awareness of lifestyle disorders and yoga’s preventive potential aligns with perceived severity and benefits. Readiness to practice and perceived obstacles, such as time constraints, sociocultural discomfort, or lack of facilities, map onto perceived barriers and self-efficacy. Using this framework allows for a deeper interpretation of the findings and offers direction for targeted interventions to promote sustained behavioral change through yoga.

This study was conceptualized to bridge this knowledge and practice gap. By focusing on MBBS students in India, this research aims to evaluate their awareness of yoga, their perceptions of its role in managing lifestyle disorders, their readiness to practice yoga regularly, and the barriers they face. Additionally, it captures student perspectives on incorporating daily yoga practice into the MBBS program, providing insights that can inform curriculum planning, student wellness initiatives, and broader policy measures for holistic medical education.

This objective aligns with recommendations from the National Task Force on Mental Health and Well-Being of Medical Students (2024), which emphasized promoting yoga as a non-pharmacological intervention to reduce stress, enhance resilience, and prevent mental illness [7]. The report advocates structured yoga modules in orientation programs and continued well-being activities to foster a positive, inclusive educational environment. Integrating yoga is not merely a wellness trend but a public health imperative, backed by national policy.

## Materials and methods

Study design and setting

This was a descriptive, cross-sectional, questionnaire-based study conducted at the All India Institute of Medical Sciences (AIIMS), Nagpur. The study targeted MBBS students from all professional years, including interns, enrolled between the academic admission years 2019 and 2023. Convenience sampling was employed, wherein all eligible students present during the data collection period were invited to participate. Participation was voluntary. Students who provided informed consent and submitted complete responses were included in the study.

Ethical considerations

The study received clearance from the Institutional Ethics Committee of AIIMS Nagpur (IEC/Pharmac/2025/1168, dated 11/01/2025). The protocol adhered to the ethical principles outlined in the Declaration of Helsinki. Confidentiality was ensured throughout the data collection and analysis process.

Tool for data collection

A structured, self-administered questionnaire was developed specifically for this study (see Appendix A). The instrument consisted predominantly of closed-ended items rated on a 5-point Likert scale, with a few multiple-selection questions related to perceived barriers and a single open-ended item to capture additional student perspectives. Questionnaire development was informed by a comprehensive review of literature on yoga adoption and health behavior among university and healthcare students.

Content validity was established through a one-round Delphi review involving three subject-matter experts from the fields of physiology, yoga therapy, and medical education, who evaluated each item for relevance, clarity, and redundancy. The draft questionnaire was then pilot-tested among 20 MBBS students who were not included in the main study sample. Based on their feedback, minor revisions were made to enhance brevity and clarity. The tool demonstrated excellent internal consistency, with Cronbach’s α scores ranging from 0.887 to 0.979 across domains.

The tool consisted of five major sections: (1) demographics, (2) awareness about yoga, (3) perceptions regarding yoga’s preventive and therapeutic role in lifestyle disorders, (4) readiness and willingness to practice yoga, and (5) perceived barriers and preferences for curriculum integration. The questionnaire included both closed-ended and Likert-scale questions.

Structure of the questionnaire

The questionnaire included the following five thematic sections:

Awareness of Yoga (10 Items)

Measured on a 5-point Likert scale (strongly disagree to strongly agree). Items assessed knowledge about yoga's role in mental clarity, stress reduction, sleep, physical health, and its general applicability.

Perceptions of Lifestyle Disorders and Benefits of Yoga (10 Items)

Measured on a 5-point Likert scale (strongly disagree to strongly agree), assessed beliefs regarding causes of lifestyle disorders and yoga’s preventive/therapeutic potential, also included perceptions of its academic and social benefits.

Readiness to Integrate Yoga Into Daily Life (8 Items)

Measured on a 5-point Likert scale (strongly disagree to strongly agree), evaluated students’ motivation, resource availability, and self-efficacy toward daily yoga.

Barriers to Yoga Practice (Multiple Choice)

Explored challenges including time constraints, lack of space or energy, motivation, guidance, and perceptions of yoga’s effectiveness.

Views on Integrating Daily Yoga Practice Into the MBBS Curriculum (9 Items)

Included preferences on session frequency, duration, delivery mode, and whether it should be mandatory or optional.

An open-ended question was also included to capture qualitative feedback about yoga and its integration into students’ daily lives.

Data collection procedure

The final questionnaire was converted into a Google Form (Google, Inc., Mountain View, CA, USA) and disseminated electronically via batch-specific WhatsApp (Meta, Menlo Park, CA, USA) groups to ensure wide outreach. Students were requested to complete the form within one week.

Data analysis

Data were cleaned and pre-processed using Python (Jupyter Notebook; Project Jupyter, USA). Descriptive statistics (frequencies, percentages, means, standard deviations) were computed for all relevant variables. Subscale scores for awareness, perception, and readiness were calculated. Internal consistency of subdomains was assessed using Cronbach’s alpha.

Jamovi software (version 2.3.28; The Jamovi Project, Sydney, Australia) was used to perform inferential analysis. As the data were non-normally distributed, non-parametric tests such as the Kruskal-Wallis H test and the Mann-Whitney U test were employed to compare scores across different batches and gender groups. A p-value of less than 0.05 was considered statistically significant.

Scoring and interpretation

Each item in the awareness, perception, and readiness sections was scored on a 5-point Likert scale (1 = strongly disagree to 5 = strongly agree). Item-wise scores were summed to yield a total score per section. The total score was then converted to a percentage score using the formula:

\[ \left( \frac{\text{Total Score}}{\text{Maximum Possible Score}} \right) \times 100 \]

Data analysis by section

Awareness, Perception, and Readiness Scores

Mean scores and their percentage were computed for each subscale. Kruskal-Wallis tests were used to identify significant differences between academic years, and Mann-Whitney U tests were applied for gender comparisons.

Barriers

Frequency analysis was performed to identify the most commonly reported obstacles. Responses were visualized using bar graphs.

Integration Preferences

Aggregated and displayed as percentages to identify the most preferred modes, durations, and timings for yoga sessions.

Qualitative Feedback

Open-ended responses were analyzed thematically to identify recurrent themes and unique student suggestions.

## Results

Participant characteristics

Using convenience sampling, a total of 159 MBBS students voluntarily responded to the questionnaire. After excluding four responses due to non-consent or incomplete data, the final analyzed sample comprised 155 (100%) participants.

The highest participation was from first-year MBBS students, comprising 89 students (57.4%), including 69 males (44.5%) and 20 females (12.9%). This was followed by second-year students with 33 participants (21.3%), consisting of 19 males (12.3%) and 14 females (9.0%). In the third year, there were 10 participants (6.5%), with seven males (4.5%) and three females (1.9%). Final-year students included 11 participants (7.1%), with nine males (5.8%) and two females (1.3%). The internship group had 12 participants (7.7%), comprising nine males (5.8%) and three females (1.9%). Overall, the gender distribution consisted of 113 males (72.9%) and 42 females (27.1%).

Awareness of yoga

The average awareness mean score among the 155 (100%) participants was 4.36, standard deviation (SD) = 0.44, with high internal consistency of subscales (Cronbach’s α = 0.856). Most students agreed or strongly agreed that yoga improves flexibility, physical strength (mean = 4.54), and overall health (mean = 4.63). All awareness-related items violated normality, prompting the use of non-parametric tests (Table 1).

**Table 1:** Awareness of Yoga and Its Perceived Health Benefits Among MBBS Students: Descriptive Statistics and Group-Wise Comparisons Mean and standard deviation (SD) values represent students’ responses on a 5-point Likert scale (1 = strongly disagree to 5 = strongly agree) for each yoga awareness item. Gender-wise comparisons were performed using the Mann-Whitney U test, and batch-wise comparisons were analyzed using the Kruskal-Wallis H test. The Kruskal-Wallis H statistic is reported as χ². A p-value < 0.05 was considered statistically significant.

Level of Yoga Awareness Items	Mean	SD	Gender-Wise Comparison		Batch-Wise Comparison	
			Statistic	P-value	χ²	P-value
Familiar with yoga	3.46	0.948	2163	0.371	7.84	0.097
Yoga can enhance mental clarity and focus	4.48	0.617	2251	0.577	1.97	0.741
Yoga can increase mindfulness and emotional resilience	4.48	0.677	2316	0.793	4.57	0.334
Yoga can reduce stress and anxiety	4.45	0.615	2336	0.869	3.08	0.544
Yoga can improve sleep quality	4.50	0.607	2278	0.664	3.14	0.534
Yoga can improve flexibility and physical strength	4.54	0.606	2351	0.918	1.40	0.845
Yoga can enhance lung function and reduce stress	4.28	0.777	2345	0.904	4.01	0.404
Yoga is beneficial for cardiovascular health	4.45	0.616	2248	0.572	3.60	0.463
Yoga is beneficial for your overall health	4.63	0.510	2304	0.740	1.63	0.803
Yoga can be practiced by all ages and fitness levels	4.54	0.606	2197	0.413	2.12	0.713
Mean yoga awareness score	4.36	0.44	-	-	-	-
Total yoga awareness score	43.80645	-	-	-	-	-
Percentage yoga awareness score	87.6129	-	-	-	-	-

**Figure 1:**
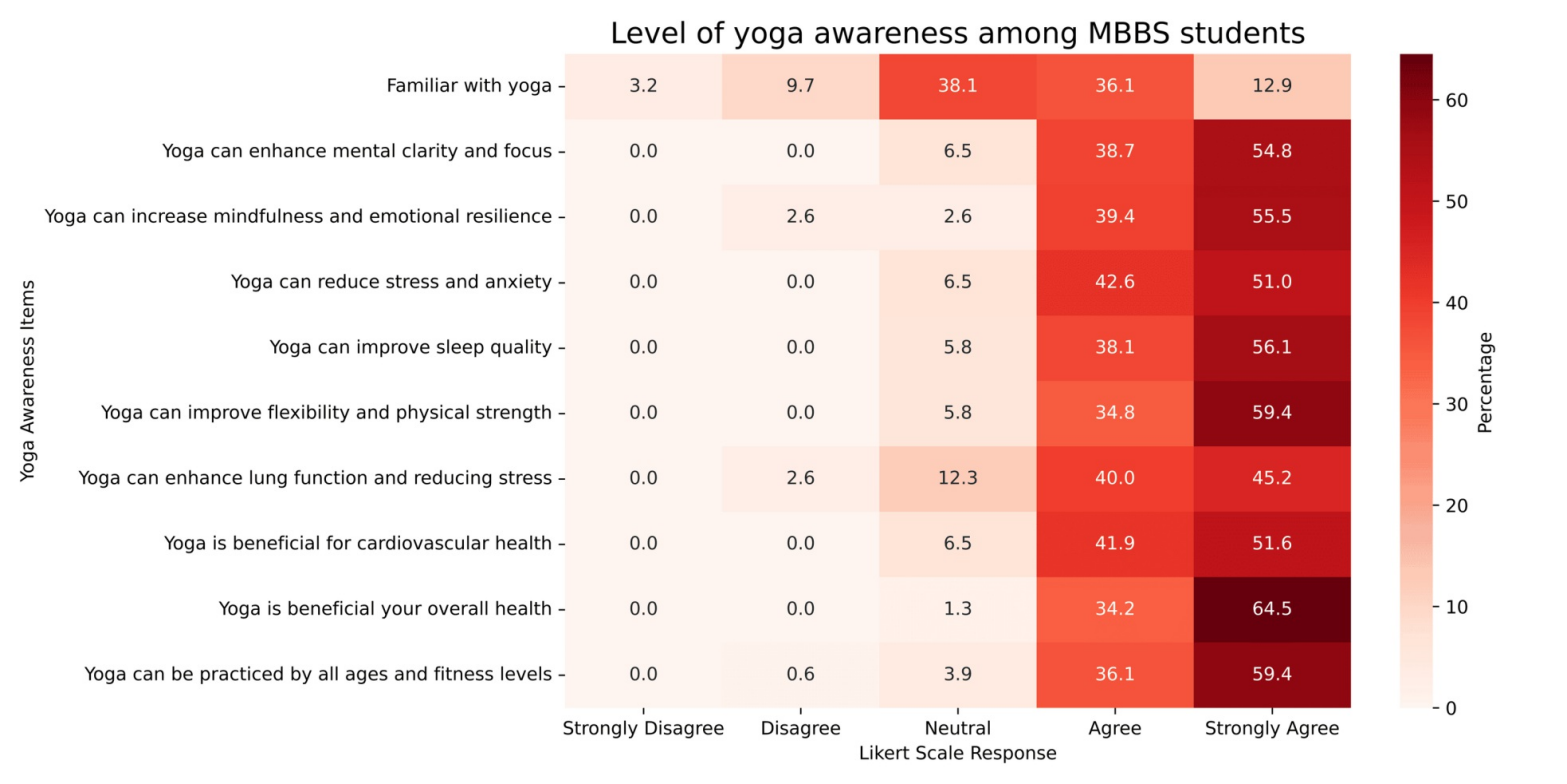
Heatmap Displaying the Level of Yoga Awareness Among MBBS Students Based on Their Likert Scale Responses Heatmap showing the distribution of MBBS students’ responses to various yoga awareness items on a 5-point Likert scale ranging from “strongly disagree” to “strongly agree.” Each cell represents the percentage of students selecting a particular response. The intensity of color corresponds to the proportion of agreement, with darker shades indicating higher agreement.

The analysis of individual awareness items reveals that students demonstrated a high level of understanding of yoga’s health benefits, with most mean scores exceeding 4.4 on a 5-point Likert scale. The highest-rated item was “Yoga is beneficial for your overall health” (mean = 4.63, SD = 0.510), closely followed by “Yoga can improve flexibility and physical strength” (mean = 4.54). These findings suggest a strong recognition of yoga’s general health-promoting properties (Figure 1).

Conversely, the lowest mean score was observed for “Familiar with yoga” (mean = 3.46, SD = 0.948), indicating that while students are well aware of the benefits, they may lack in-depth familiarity or practical engagement with yoga itself.

The total sum of the mean values across all 10 awareness items was 43.80, which translates to a percentage mean of 87.6% when scaled appropriately. This reflects an overall high level of agreement with yoga-related awareness statements among participants.

No statistically significant gender-wise or batch-wise differences were found for any of the awareness items (p > 0.05). However, the item “Familiar with yoga” showed a trend toward significance across academic years (p = 0.097), hinting that exposure to yoga may slightly increase with advancement through the MBBS program.

Perceptions of lifestyle disorders and the benefits of yoga

The total mean perception score across all 10 items was 42.01 out of 50, which corresponds to a percentage mean of 84.03%. The mean score for perceptions about the role of yoga in managing lifestyle disorders was 4.20, SD = 0.55, with good reliability (Cronbach’s α = 0.888). Students overwhelmingly endorsed yoga as an effective preventive and therapeutic modality (Table 2).

**Table 2:** Perceptions of Lifestyle Disorders and the Perceived Benefits of Yoga Among MBBS Students: Descriptive Statistics and Group-Wise Comparisons Mean and standard deviation (SD) values represent MBBS students’ responses to each item on a 5-point Likert scale (1 = strongly disagree to 5 = strongly agree). Gender-wise comparisons were analyzed using the Mann-Whitney U test, while batch-wise comparisons were conducted using the Kruskal-Wallis H test, which is reported as χ². P-values < 0.05 were considered statistically significant.

Perceptions of Lifestyle Disorders and the Benefits of Yoga Items	Mean	SD	Gender-Wise Comparison		Batch-Wise Comparison	
			Statistic	P-value	χ²	P-value
Awareness of lifestyle disorders	4.02	0.901	2065	0.188	24.351	<0.001
Lifestyle disorders are caused by unhealthy habits	4.41	0.728	2363	0.964	4.637	0.327
Lifestyle disorders can be effectively prevented through regular physical activity	4.42	0.643	2324	0.827	4.907	0.297
Yoga is an effective way to prevent lifestyle disorders	4.27	0.724	2357	0.946	5.512	0.239
Lifestyle disorders can be reversed or significantly improved through consistent yoga practice	4.15	0.788	2338	0.879	0.841	0.933
Yoga should be recommended as a complementary therapy for managing lifestyle disorders	4.28	0.689	2308	0.771	9.449	0.051
Yoga can help reduce dependence on medication for lifestyle disorders	4.17	0.731	2235	0.542	1.307	0.860
Mental and emotional benefits of yoga are crucial in managing stress-related lifestyle disorders	4.31	0.717	2210	0.468	3.705	0.447
Daily yoga would help improve the academic performance of MBBS students	4.01	0.886	2123	0.285	1.584	0.812
Daily yoga could help in creating social connectivity among MBBS students	3.97	0.932	2315	0.803	1.182	0.881
Mean perception score	4.20	0.55	-	-	-	-
Total perception score	42.0129	-	-	-	-	-
Percentage perception score	84.02581	-	-	-	-	-

**Figure 2:**
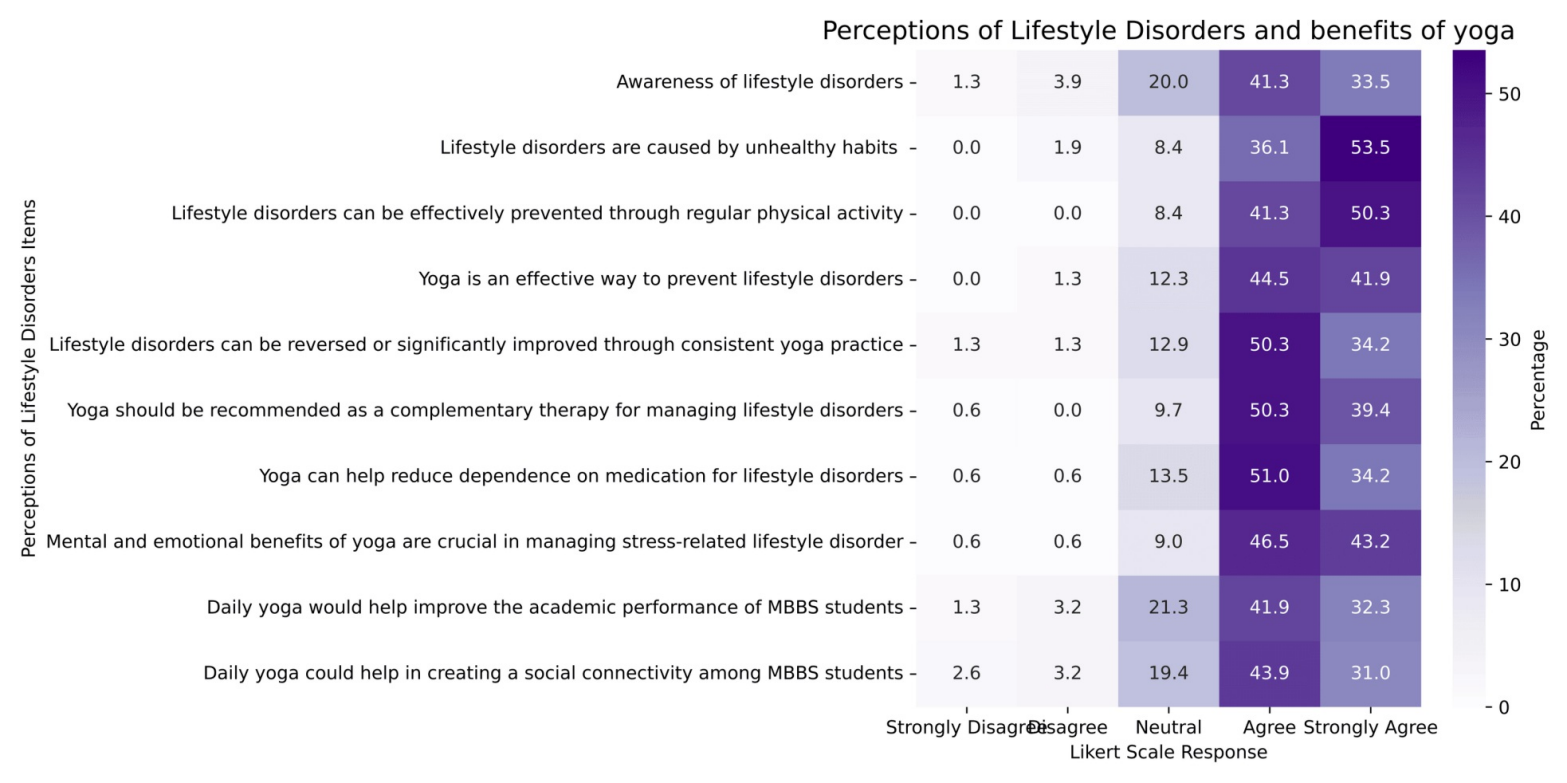
Perceptions of Lifestyle Disorders and the Perceived Benefits of Yoga Among MBBS Students Heatmap showing MBBS students’ perceptions regarding lifestyle disorders and the perceived benefits of yoga. Each item was rated on a 5-point Likert scale from “strongly disagree” to “strongly agree.” Percentages within each cell represent the proportion of students who selected each response. Darker shades correspond to higher percentages, indicating stronger agreement.

Among the 10 items measuring perceptions related to yoga and lifestyle disorders, the highest mean scores were for “Lifestyle disorders are caused by unhealthy habits” (mean = 4.41, SD = 0.728) and “Lifestyle disorders can be effectively prevented through regular physical activity” (mean = 4.42, SD = 0.643), reflecting strong student agreement with foundational concepts in lifestyle medicine. The statement “Daily yoga could help in creating social connectivity among MBBS students” received the lowest mean score (mean = 3.97, SD = 0.932), suggesting relatively lower endorsement of yoga’s social impact (Figure 2).

No statistically significant gender-wise differences were observed for any item (p > 0.05).

The item “Awareness of lifestyle disorders” showed a statistically significant difference across MBBS batches (χ² = 24.35, df = 4, p < 0.001) with a moderate effect size (ε² = 0.1581). Post hoc pairwise analysis using the Dwass-Steel-Critchlow-Fligner test revealed that this difference was primarily driven by significantly higher scores for second-year students compared to first-year students (W = 4.991, p = 0.004), and interns compared to first-year students (W = 5.003, p = 0.004). These findings suggest that as students progress through the MBBS program, their understanding of lifestyle disorders and their link to behavior and preventive strategies, such as yoga, improves notably. There were no statistically significant differences among other batches.

The item “Yoga should be recommended as complementary therapy for managing lifestyle disorders” showed a borderline significant difference across batches (χ² = 9.45, df = 4, p = 0.051), with a small effect size (ε² = 0.0614). Pairwise comparisons showed significantly higher scores among interns compared to first-year (p = 0.004) and second-year (p = 0.004) students, suggesting that increased clinical exposure and maturity in medical training may positively shape students’ views about yoga’s relevance to lifestyle disorder prevention and therapy.

Readiness to integrate daily yoga practice

The total mean readiness score across all eight items was 27.29 out of 40, corresponding to a percentage mean of 68.22%. The average item score was 3.41 (SD = 0.872), with good internal consistency (Cronbach’s α = 0.905). While students acknowledged the value of yoga, consistent motivation and confidence in maintaining regular practice were notably lacking (Table 3).

**Table 3:** Readiness to Integrate Yoga Practice Among MBBS Students: Descriptive Statistics and Group-Wise Comparisons Mean and standard deviation (SD) values represent MBBS students’ responses to each item on a 5-point Likert scale (1 = strongly disagree to 5 = strongly agree). Gender-wise comparisons were conducted using the Mann-Whitney U test, and batch-wise comparisons were analyzed using the Kruskal-Wallis H test, reported as χ². A p-value < 0.05 was considered statistically significant.

Readiness to Integrate Yoga Items	Mean	SD	Gender-Wise Comparison		Batch-Wise Comparison	
			Statistic	P-value	χ²	P-value
Ready to prioritize yoga practice	3.46	1.071	2171	0.399	4.34	0.363
Wake up early for yoga practice	3.21	1.252	2120	0.297	4.86	0.302
Motivated for daily yoga practice	3.41	1.126	2178	0.414	2.56	0.633
Have the necessary resources to practice yoga	3.22	1.234	2009	0.132	7.85	0.097
Willing to seek guidance from a yoga instructor	3.41	1.167	2317	0.817	8.28	0.082
Prepared to handle any physical challenges	3.53	0.982	1874	0.035	2.83	0.587
Believe that the benefits of yoga outweigh the effort required to practice it daily	3.81	0.966	2217	0.509	5.21	0.266
Feel confident to stay committed over the long term	3.24	1.111	2126	0.305	1.73	0.785
Mean readiness score	3.41	0.872	-	-	-	-
Total readiness score	27.29	-	-	-	-	-
Percentage readiness score	68.22	-	-	-	-	-

**Figure 3:**
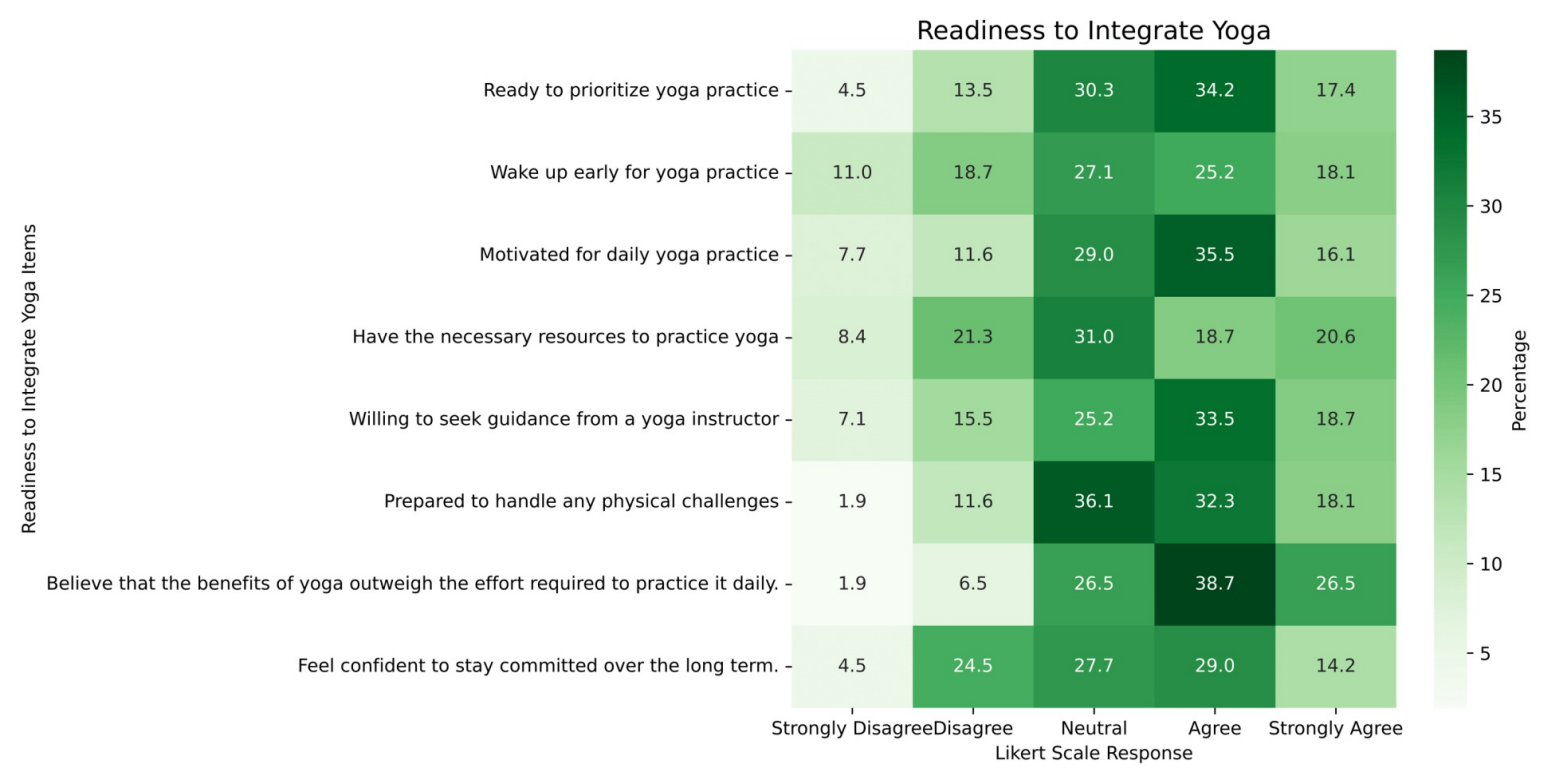
Readiness to Integrate Yoga Practice Among MBBS Students Based on Likert Scale Responses Heatmap depicting MBBS students’ readiness to integrate yoga into their daily routine. Each item was rated on a 5-point Likert scale from “strongly disagree” to “strongly agree.” Cell values represent the percentage of students selecting each response, with darker shades indicating higher agreement.

Among the items, “Believe that the benefits of yoga outweigh the effort required to practice it daily” received the highest mean score (mean = 3.81, SD = 0.966), suggesting a positive outlook toward yoga’s value. In contrast, “Wake up early for yoga practice” (mean = 3.21, SD = 1.252) and “Have the necessary resources to practice yoga” (mean = 3.22, SD = 1.234) scored the lowest, indicating logistical and routine-related barriers (Figure 3).

A statistically significant gender-based difference was observed in the item “Prepared to handle any physical challenges” (W = 1874, p = 0.035), where males (mean = 3.63) rated themselves higher than females (mean = 3.26), suggesting perceived differences in physical readiness. However, no significant batch-wise differences were observed across any readiness items (p > 0.05).

Overall, the findings indicate that while students generally acknowledge the benefits of yoga, their readiness to integrate it into daily life is moderate, affected primarily by time, resources, and physical self-efficacy.

Barriers to yoga practice

The most frequently reported barriers included time constraints (112, 72.26%), lack of consistency (93, 60.00%), and unmotivation to wake up early (83, 53.55%), indicating that behavioral and routine-related challenges are the most dominant obstacles. Other issues cited were the need for guidance (63, 40.65%) and the lack of space (42, 27.10%). Only eight (5.16%) found yoga physically challenging, and 17 (10.97%) perceived it as less effective than other forms of exercise.

When comparing male and female respondents, a higher proportion of females reported several barriers. Notably, 83.33% of females cited time constraints compared to 68.14% of males, though this difference did not reach statistical significance (p = 0.07). Similarly, a greater proportion of females reported difficulty in maintaining consistency (69.05%) than males (56.64%), but the difference was not significant (p = 0.20).

The only barrier that demonstrated a statistically significant gender difference was physical tiredness, reported by 28.57% of females compared to 11.50% of males (p = 0.01), indicating that females were more likely to perceive physical fatigue as a hindrance to yoga practice.

Other barriers, such as lack of a quiet space, perception of yoga as boring or less effective, and perceiving yoga as physically challenging, did not differ significantly between the two genders (p > 0.05 for all) (Table 4).

**Table 4:** Gender-Wise Distribution of Reported Barriers to Yoga Practice Among MBBS Students “N” indicates the number of respondents endorsing each barrier within each gender group, while "%" denotes the percentage of respondents who endorsed each barrier. Percentages are calculated using the total number of respondents in each group as the denominator - N = 155 overall, with males = 113 and females = 42, each group considered as 100%.

Barrier Option	Total Respondents, N (%)	Males, N (%)	Females, N (%)	P-value
Time constraints	112 (72.26)	77 (68.14)	35 (83.33)	0.07
Difficulty in maintaining consistency	93 (60.00)	64 (56.64)	29 (69.05)	0.2
Unmotivated to wake up early	83 (53.55)	58 (51.33)	25 (59.52)	0.47
Need for guidance	63 (40.65)	45 (39.82)	18 (42.86)	0.85
Lack of quiet and comfortable space	42 (27.10)	29 (25.66)	13 (30.95)	0.54
Yoga is perceived as boring	26 (16.77)	20 (17.70)	6 (14.29)	0.81
Physical tiredness	25 (16.13)	13 (11.50)	12 (28.57)	0.01
Yoga is perceived as less effective than other exercises	17 (10.97)	15 (13.27)	2 (4.76)	0.16
Yoga is physically challenging	8 (5.16)	7 (6.19)	1 (2.38)	0.68

Environmental and perceptual barriers were reported less frequently. These included a lack of a quiet and comfortable space (29 students; 27.5% overall - 29 males (26.1%) and 13 females (30.9%)) and the perception that yoga is boring (20 students; 16.9% overall - 20 males (18.0%) and 6 females (14.3%)).

Physical limitations were less commonly cited, including physical tiredness (15 (16.3%); 15 (13.5%) males and 2 (4.8%) females), the belief that yoga is less effective than other forms of exercise (13 (11.1%); 13 (11.7%) males and 12 (28.6%) females), and the belief that yoga is physically challenging (8 (5.2%); 7 (6.3%) males and 1 (2.4%) female).

These findings suggest that logistical and motivational issues are the most significant barriers to regular yoga practice in this cohort, particularly among female students, while physiological deterrents were less prominent.

Gender-wise comparison of barriers to yoga practice

**Figure 4:**
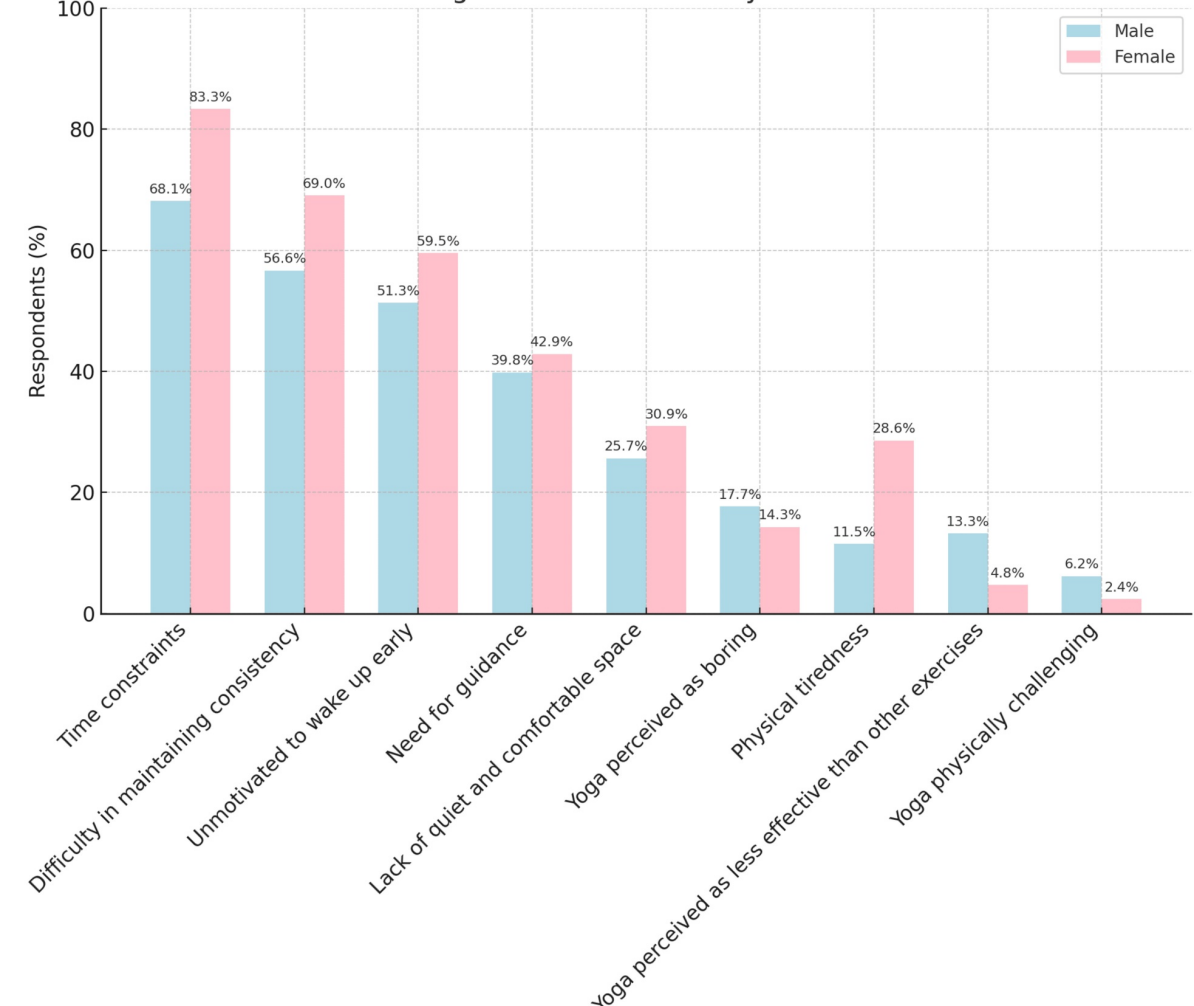
Gender-Wise Comparison of Reported Barriers to Yoga Practice Among MBBS Students Bar chart illustrating the percentage of male and female MBBS students reporting specific barriers to regular yoga practice. Each barrier is displayed along the x-axis, with corresponding response percentages on the y-axis.

Figure 4 depicts the proportion of male (light blue bars) and female (pink bars) respondents who endorsed each of nine predefined barriers to practicing yoga (N = 155; males = 113, females = 42). Taken together, the figure highlights that while most external barriers (time, consistency, early rising) are common to both genders, fatigue uniquely affects female participants and may warrant targeted scheduling, recovery, or motivational strategies in programmes designed to improve yoga adherence (Figure 4).

Views on integrating daily yoga practice into the MBBS curriculum

Students reported a generally positive attitude toward the integration of daily yoga practice into the MBBS curriculum, with an overall mean integration score of 3.93 (on a 5-point scale) and a percentage score of 78.54%, indicating high agreement levels (Table 5).

**Table 5:** Student Views on Integrating Daily Yoga Practice Into the MBBS Curriculum: Descriptive Statistics and Group-Wise Comparisons Mean and standard deviation (SD) values reflect MBBS students’ responses to each item on a 5-point Likert scale (1 = strongly disagree to 5 = strongly agree). Gender-wise comparisons were performed using the Mann-Whitney U test, while batch-wise comparisons were analyzed using the Kruskal-Wallis H test, reported as χ².

Views on Integrating Yoga Practice Into the MBBS Curriculum Items	Mean	SD	Gender-Wise Comparison		Batch-Wise Comparison	
			Statistic	P-value	χ²	P-value
Daily yoga sessions should be part of the MBBS curriculum	3.66	1.19	2236	0.567	7.83	0.098
Yoga sessions should include an educational component to explain the health benefits of each practice	3.97	1.03	1803	0.016	6.11	0.191
Improvement in students' physical and mental health should be assessed periodically	4.15	0.938	2117	0.269	4.09	0.393
Mean integration score	3.93	0.824	-	-	-	-
Total integration score	11.78	-	-	-	-	-
Percentage integration score	78.53	-	-	-	-	-

Among the individual items, the highest agreement was observed for the view that physical and mental health should be assessed periodically (mean = 4.15, SD = 0.94), suggesting strong student support for incorporating regular wellness assessments as part of academic life. This item also showed no statistically significant differences across gender (U = 2117, p = 0.269) or MBBS batches (χ² = 4.09, p = 0.393), indicating broad consensus across subgroups.

**Figure 5:**
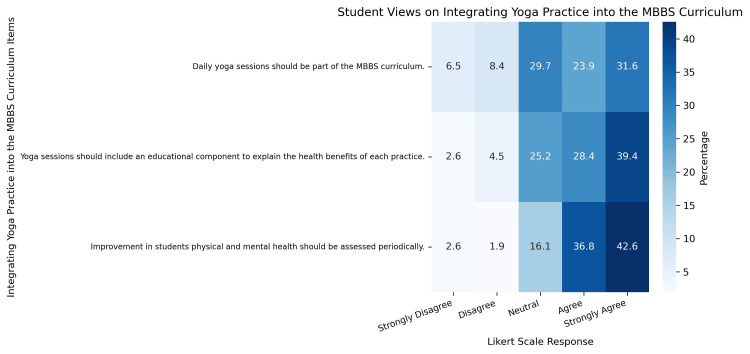
Heatmap Illustrating MBBS Students’ Perspectives on Integrating Yoga Practice Into the Medical Curriculum Heatmap showing MBBS students’ responses on integrating yoga practice into the medical curriculum, rated on a 5-point Likert scale from “strongly disagree” to “strongly agree.” Each cell represents the percentage of students who selected that response. The darkest shades reflect the highest agreement.

The inclusion of an educational component within yoga sessions also received strong endorsement (mean = 3.97, SD = 1.03), with a statistically significant gender difference (U = 1803, p = 0.016), suggesting that female students were more inclined to value informative content within yoga practice. However, batch-wise differences were not statistically significant (χ² = 6.11, p = 0.191) (Figure 5).

While the idea of daily yoga sessions also received moderate agreement (mean = 3.66, SD = 1.19), no significant differences were observed across gender (U = 2236, p = 0.567) or academic year (χ² = 7.83, p = 0.098), though a near-significant trend was noted across batches.

These findings reflect a generally favorable perception of integrating yoga into the medical curriculum, with emphasis on wellness monitoring and educational reinforcement within yoga sessions.

Preference for voluntary participation in yoga sessions

When asked about their preference regarding participation in yoga sessions, a majority of students (107, 69.5%) indicated that sessions should be optional, while 47 (30.5%) favored mandatory inclusion. This suggests that while students generally support the integration of yoga into the curriculum, most prefer flexibility in participation, possibly due to individual schedules, interest levels, or perceived relevance. The data highlights the importance of offering structured yet voluntary opportunities for engagement to ensure broad acceptance and sustained involvement.

Student preferences for yoga session timing

When asked about preferred scheduling for yoga sessions, 114 (73.55%) students opted for flexible timings, including 80 (70.18%) males and 34 (29.82%) females, allowing them to choose time slots that best suited their schedules. A substantial portion, 66 (42.58%) - comprising 46 (69.70%) males and 20 (30.30%) females - preferred sessions to be conducted early in the morning before academic classes, suggesting openness to integrating yoga into the start of the academic day. Scheduling yoga between 8 AM and 5 PM during academic hours was favored by 38 (24.52%) students, including 26 (68.42%) males and 12 (31.58%) females. A minority, 24 (15.48%) - with 20 (83.33%) males and four (16.67%) females - preferred evening sessions after classes. These findings underscore the importance of flexibility and personalization in scheduling, as rigid timing may limit participation despite a generally positive attitude toward yoga integration.

Preferred duration of yoga sessions among students

When asked about the ideal duration for yoga sessions, the majority of students (93, 61.59%) preferred 20-30 minute sessions, including 63 (67.74%) males and 30 (32.26%) females, indicating a clear preference for a brief but structured practice. A smaller proportion - 30 (19.87%) students, 25 (83.33%) males and five (16.67%) females - favored short sessions lasting 10-15 minutes. Meanwhile, 24 (15.89%) students preferred a more extended session of 45-60 minutes, comprising 18 (75.0%) males and six (25.0%) females. Only four (2.65%) students were in favor of sessions lasting 60 minutes or more, including three (75.0%) males and one (25.0%) female. These findings suggest that compact, time-efficient yoga sessions are generally more acceptable to students, likely due to their alignment with tight academic schedules and limited availability.

Preferred weekly frequency of yoga practice

When asked about their preferred frequency for yoga sessions, the most common choice was three days a week, selected by 63 (40.65%) students, including 44 (69.84%) males and 19 (30.16%) females. This was followed by one day a week, chosen by 32 (20.65%) students - 27 (84.38%) males and five (15.62%) females. Both daily sessions and five days a week were each preferred by 23 (14.84%) students. Among those who selected daily practice, 17 (73.91%) were males and six (26.09%) were females, while the five-day option included 15 (65.22%) males and eight (34.78%) females. The least preferred option was two days a week, selected by 21 (13.55%) students, with 15 (71.43%) males and six (28.57%) females.

The preferred frequency for yoga practice among students showed a clear leaning toward moderate engagement. A significant proportion of students opted for three days a week (63, 40.65%), suggesting that a structured yet flexible routine is most acceptable in the academic setting. Lighter commitments, such as one day a week (32, 20.65%) or two days a week (21, 13.55%), were also chosen by some, likely reflecting either time constraints or a cautious interest in yoga. Notably, only a smaller fraction expressed willingness for daily (23, 14.84%) or five-day-per-week (23, 14.84%) practice, highlighting that intensive schedules may not align with the majority’s current lifestyle or readiness. Across all frequency options, male students consistently represented a larger share, suggesting either higher participation or greater interest in yoga across varied schedules.

These findings highlight a general inclination toward moderate, structured frequency in yoga sessions, with flexibility preferred over daily commitment due to academic schedules.

Preferred mode of yoga session delivery

In terms of preferred formats for yoga practice, the majority of students (116, 74.84%) opted for physical classes, with 81 (69.83%) males and 35 (30.17%) females selecting this mode. Online pre-recorded video sessions were chosen by 57 (36.77%) students, including 42 (73.68%) males and 15 (26.32%) females. Meanwhile, online live sessions with a qualified instructor were preferred by 26 (16.77%) students, of whom 17 (65.38%) were males and nine (34.62%) were females. These findings emphasize that while some flexibility is appreciated, students overwhelmingly favor traditional in-person formats for yoga, possibly due to the experiential and guided nature of the practice.

Preferred focus areas within yoga sessions

Based on the students’ preferences for yoga content, meditation was the most commonly selected component, reported by 131 (84.52%) students, with 97 (74.05%) males and 34 (25.95%) females. This was followed by breathing exercises, chosen by 119 (76.77%) students - 85 (71.43%) males and 34 (28.57%) females. Physical postures were preferred by 117 (75.48%) students, including 81 (69.23%) males and 36 (30.77%) females. Lastly, 113 (72.90%) students indicated a focus on stress relief, comprising 76 (67.26%) males and 37 (32.74%) females. These findings suggest that while meditation and stress relief were slightly more popular, all four components were well-represented, indicating broad interest in diverse aspects of yoga across both genders.

Thematic analysis of open-ended responses

A total of 142 open-ended responses were received from MBBS students regarding their views on yoga and its integration into daily life. Several responses included multiple sentiments, resulting in 167 coded thematic references. Thematic analysis revealed the following seven key themes, each accompanied by representative insights and their proportion out of the total:

Theme 1: Perceived Physical and Mental Health Benefits of Yoga - 45 (26.95%)

Students frequently acknowledged yoga as a valuable practice that improves flexibility, posture, physical fitness, mental clarity, and overall well-being. Many described yoga as “very effective,” “good for health,” or “essential for maintaining a healthy lifestyle.” Some emphasized its holistic nature, benefiting both body and mind.

Theme 2: Stress Relief and Emotional Balance - 35 (20.96%)

Yoga was widely recognized as a tool for managing stress, calming the mind, and promoting emotional stability. Respondents noted that yoga helped reduce anger, improved focus, and enabled them to handle academic stress more effectively. Comments highlighted yoga’s role in cultivating peace, calmness, and mindfulness.

Theme 3: Challenges to Regular Practice - Time, Motivation, and Consistency - 30 (17.96%)

Several participants reported practical barriers to incorporating yoga into their routines, including a lack of time, motivation, or difficulty in maintaining consistency. Comments such as “time is the problem,” “I lack the motivation,” and “execution is difficult unless made part of my schedule” reflect the struggle to prioritize yoga amidst academic responsibilities.

Theme 4: Suggestions for Integration Into the Medical Curriculum - 25 (14.97%)

A significant number of students recommended formal integration of yoga sessions within the MBBS curriculum. They suggested weekly structured yoga classes, group sessions, or dedicated time slots to facilitate regular practice. Respondents believed that institutional support would help normalize yoga as a core component of student life.

Theme 5: Cultural and Spiritual Significance - 10 (5.99%)

Some responses reflected the cultural and spiritual importance of yoga, especially among students with prior exposure through spiritual retreats or traditional teachings. These participants advocated for preserving the heritage of yoga and promoting awareness of its roots in Indian culture and philosophy.

Theme 6: Need for Awareness and Proper Guidance - 15 (8.98%)

A subset of students expressed interest in yoga but cited a lack of awareness, technical knowledge, or proper guidance as limiting factors. They reported difficulties in understanding certain practices and emphasized the need for trained instructors and accessible information on yoga techniques.

Theme 7: Critical or Alternative Perspectives - 7 (4.19%)

A small group of students expressed skepticism about yoga, viewing it as less engaging compared to other forms of exercise, like sports or strength training. Some considered yoga to be time-intensive with limited physical benefits, while others questioned its relevance or sustainability.

This thematic analysis highlights both the enthusiasm and the barriers students experience in yoga practice. The insights gained underscore the importance of structured support, guided implementation, and contextual sensitivity in designing yoga-related interventions for medical students.

## Discussion

This study provides a comprehensive examination of MBBS students’ awareness, perceptions, readiness, and practical challenges related to yoga, along with their preferences regarding its integration into the medical curriculum. The findings reveal a noteworthy paradox - while awareness of yoga’s benefits is high (mean awareness score = 87.6%) and perceptions of its role in managing lifestyle disorders are strongly positive (mean = 84.0%), actual readiness to practice yoga regularly remains moderate (68.2%). This disconnect between knowledge and practice reflects a common trend in health behavior, where awareness does not necessarily translate into sustained behavioral change without enabling environments and structured interventions. This is consistent with earlier research, including that by Shrestha et al., who noted that while 96.6% of medical students knew yoga's benefits, only 14.2% practiced it regularly [8]. Mishra et al. reported that although 91.8% of students acknowledged the importance of yoga, only 18.4% practiced it consistently [9].

High mean scores for items relating to yoga’s impact on flexibility, mental clarity, and sleep quality support the growing body of literature emphasizing yoga’s role in physical and psychological well-being among healthcare students [3-5,8-11]. However, the relatively low score on being “familiar with yoga” and readiness to “wake up early” reflects the logistical and motivational challenges students face, despite understanding the benefits [12,13].

Gender-based differences were also noted. Confidence in handling physical challenges was reported by 63 (55.8%) males, compared to 16 (38.1%) females. On the other hand, 27 (64.3%) females preferred inclusion of educational components in yoga sessions, compared to 53 (46.9%) males, suggesting that males and females may respond differently to motivational cues, and programs must account for this variation [14,15].

Batch-wise trends also revealed increasing awareness with academic exposure. Awareness of lifestyle disorders was highest among second-year students at 33 (21.3%) and interns at 12 (7.7%), compared to 89 (57.4%) first-year students. This supports the hypothesis that academic and clinical maturity enhances students’ understanding of lifestyle-related health issues and the relevance of yoga [9,10]. Similar findings have been noted in studies from India and abroad, where clinical exposure appears to shape students’ readiness for holistic approaches [16,17].

Based on the gender-wise comparison of barriers to yoga practice, time constraints, difficulty in maintaining consistency, and lack of motivation to wake up early were the most frequently reported barriers across both genders. Although females consistently reported higher percentages across most barriers, only physical tiredness showed a statistically significant difference (p = 0.01), being notably more prevalent among females (28.6%) than males (11.5%). This suggests that fatigue may be a gender-specific barrier, potentially influenced by physiological or psychosocial factors, echoing the findings of other studies that have shown that time pressure, academic stress, and poor mental well-being are widespread among medical students [3,4,15,18]. These findings highlight the need for tailored interventions that consider gender-specific challenges to improve yoga participation, like flexible scheduling, and access to yoga instructors and motivational support within institutional wellness planning.

Despite these challenges, the study observed overwhelming support (140, 90.3%) for integrating yoga into the MBBS curriculum. Preferences leaned toward in-person sessions held three times a week, lasting 20-30 minutes, with flexibility in timing. Students favored content that balanced physical postures, breathing, meditation, and stress relief. These preferences provide clear, actionable insights for curriculum designers, especially in light of rising concerns about student mental health, aiming to implement holistic wellness strategies in medical education [5,7,12].

Importantly, students’ recognition of yoga’s value for academic performance, stress reduction, and social connectivity suggests that yoga is seen not merely as a fitness routine but as a comprehensive tool for personal and professional development. This aligns with recommendations from the Mental Health Task Force (2024) [7] and is further supported by Shinde et al., who demonstrated that yoga-based interventions positively influenced medical undergraduates’ perceptions and practices [10]. The positive attitudinal shift noted in recent curricular changes in India [9] and calls for structured modules abroad [12,17] further support the feasibility of institutionalizing yoga in formal learning pathways.

Qualitative analysis of open-ended responses further emphasized yoga’s role in stress management, emotional balance, and concentration. At the same time, students expressed the need for structured guidance and institutional support - echoing findings by Surendran et al., who described a peer-led “ripple effect” model. In this model, yoga-practicing students influenced their peers through mentoring, thereby significantly increasing both awareness and participation [19]. Implementing a similar peer mentorship approach within the MBBS curriculum could address motivational and logistical challenges identified in this study.

Furthermore, integrating yoga into the curriculum can legitimize its practice and equip future doctors with skills in preventive and promotive healthcare. This is particularly important given the increasing burden of stress and burnout in medical training environments, as also suggested by Surendran et al., which can legitimize its role within formal education. It can also equip future doctors with holistic, integrative approaches to preventive and promotive healthcare [19], the growing recognition of yoga as an evidence-based intervention with psychosomatic benefits [12,17].

In summary, while MBBS students clearly understand the value of yoga and show a willingness to engage, actual practice is hindered by several practical barriers. Interventions that are voluntary, brief, flexible, and supported by peer-led strategies are more likely to yield sustained engagement. Integrating yoga into the curriculum not only benefits students' well-being but also prepares future clinicians to advocate for integrative care for their patients.

Limitations

This study was conducted at a single medical institute, which may limit the generalizability of the findings to other medical colleges across diverse geographical and cultural contexts in India. The data relied on self-reported responses, which are subject to recall bias and social desirability bias. Additionally, as the study used a cross-sectional design, it captures perceptions and behaviors at a single time point and cannot assess changes over time or causality. The use of an online survey format may have excluded students less responsive to digital communication, potentially influencing participation rates and response patterns. Moreover, important sociodemographic, cultural, and religious factors that could influence yoga perceptions and practices were not assessed and should be explored in future studies.

Recommendations

To build on the findings of this study, future research should adopt a multi-centric approach, involving medical colleges from diverse regions across India to enhance the generalizability of results and capture variations in institutional culture and student demographics. Longitudinal studies are also warranted to evaluate changes in awareness, perceptions, and readiness over time, as well as the sustained impact of yoga on academic performance, mental well-being, and lifestyle behaviors throughout the MBBS course.

Curriculum development efforts should involve a dedicated committee comprising medical educators, yoga practitioners, mental health professionals, and student representatives to design structured, evidence-based yoga modules that are pedagogically sound and aligned with student needs. These modules should be flexible, preferably offered as optional wellness components with varied scheduling options (e.g., early morning, between classes, or evening) to ensure accessibility without adding to academic burden.

Additionally, institutional policies should encourage the integration of yoga within broader student wellness programs, particularly those targeting stress management and resilience-building. Motivational strategies, including peer-led sessions, mentorship models, and incentive-based participation, may help overcome key barriers such as inconsistency and lack of motivation. Gathering feedback through periodic assessments and incorporating student input will be crucial in refining session content and delivery.

Engaging stakeholders beyond students, such as faculty members and administrators, will be essential for ensuring buy-in, resource allocation, and long-term sustainability. Collectively, these efforts can support the structured inclusion of yoga in medical education, fostering a healthier, more resilient future healthcare workforce.

Implications

The findings of this study have several important implications for medical education, student wellness programs, and health policy. First, the high levels of awareness and positive perceptions toward yoga among MBBS students underscore the relevance and acceptability of yoga as a health-promoting intervention within the medical academic context. Despite moderate readiness levels, the strong endorsement for integrating structured yoga sessions into the curriculum highlights a clear demand for institutional support and formal inclusion of wellness strategies.

Second, the study reveals a critical gap between awareness and actionable behavior, suggesting that current wellness initiatives may not sufficiently address practical barriers such as time constraints, motivation, and access to guided sessions. Addressing these through tailored, flexible, and student-centered yoga programs could improve participation and foster healthier lifestyle habits during the formative years of medical training.

Third, the gender and batch-wise differences in perceptions and readiness suggest that a one-size-fits-all approach may not be effective. Instead, implementation strategies must be inclusive and responsive to the diverse needs of students across academic stages and demographic groups. This can be achieved by embedding yoga within a broader framework of mental health and lifestyle management, supported by policy-driven guidelines.

Lastly, by equipping future healthcare professionals with experiential knowledge of yoga and its therapeutic potential, medical institutions can play a pivotal role in advancing integrative and preventive healthcare models. This aligns with the growing global emphasis on holistic and non-pharmacological interventions and supports the NMC’s call for competency-based, student-friendly medical education. Thus, the study provides empirical evidence that can inform curriculum reform, institutional wellness planning, and national strategies for enhancing the well-being of medical students.

## Conclusions

This study provides comprehensive insights into MBBS students’ awareness, perceptions, readiness, and preferences regarding the integration of daily yoga practice into their daily lives and medical curriculum. While the awareness of yoga's health benefits was high (87.6%) and perceptions toward its role in managing lifestyle disorders were strongly positive (84.0%), actual readiness to incorporate yoga into daily routines was moderate (68.2%), limited by practical barriers such as time constraints, inconsistency, and lack of motivation. Despite these challenges, a substantial majority expressed strong support for integrating daily yoga practice into the MBBS curriculum, with a preference for short, flexible, in-person sessions focused on both mental and physical well-being. Gender and batch-wise comparisons revealed subtle but important differences in readiness and perceptions, highlighting the need for tailored strategies.

The findings underscore the importance of transitioning from awareness to actionable engagement by addressing logistical challenges and aligning yoga offerings with students’ schedules and interests. Integrating structured yoga modules within the medical curriculum - as optional, wellness-oriented sessions emphasizing stress relief and guided practice - can serve as a sustainable, non-pharmacological intervention to promote resilience and holistic health in future healthcare professionals.

## Appendices

Appendix A

Section A: Level of Awareness

Please respond using a 5-point Likert scale: 1 = Strongly Disagree, 2 = Disagree, 3 = Neutral, 4 = Agree, 5 = Strongly Agree

1. Are you familiar with yoga?

2. Are you aware that regular yoga practice can enhance mental clarity and focus?

3. Are you aware that yoga can increase mindfulness and emotional resilience?

4. Are you aware that yoga can help in reducing stress and anxiety?

5. Are you aware that yoga can improve sleep quality?

6. Are you aware that yoga can improve flexibility and physical strength?

7. Are you aware of breathing techniques (pranayama) in yoga for enhancing lung function and reducing stress?

8. Are you aware that yoga is beneficial for cardiovascular health?

9. Do you believe yoga can be beneficial for your overall health?

10. Are you aware that yoga can be practiced by individuals of all ages and fitness levels?

Section B: Perceptions Regarding Lifestyle Disorders and the Role of Yoga

Please respond using a 5-point Likert scale as above

11. Are you aware of lifestyle disorders?

12. Do you perceive that lifestyle disorders, such as obesity and hypertension, are primarily caused by unhealthy habits like diet, sedentary lifestyle, poor sleep, smoking, alcohol consumption, stress, and low social connectivity?

13. Do you think lifestyle disorders can be effectively prevented through regular physical activity?

14. Do you think yoga is an effective way to prevent lifestyle disorders?

15. Do you think lifestyle disorders can be reversed or significantly improved through consistent yoga practice?

16. Do you think yoga should be recommended as a complementary therapy for managing lifestyle disorders?

17. Do you perceive that yoga can help reduce dependence on medication for lifestyle disorders?

18. Do you think the mental and emotional benefits of yoga are crucial in managing stress-related lifestyle disorders?

19. Do you think daily yoga sessions would help improve the academic performance of MBBS students?

20. Do you believe that daily yoga sessions could help in creating social connectivity among MBBS students?

Section C: Readiness to Integrate Yoga Into Daily Life

Please respond using a 5-point Likert scale: 1 = Strongly Disagree, 2 = Disagree, 3 = Neutral, 4 = Agree, 5 = Strongly Agree

21. I am ready to prioritize the necessary lifestyle changes to accommodate yoga in my daily activities, despite a busy schedule.

22. I believe that I can wake up early for yoga practice.

23. I feel motivated to start practicing yoga regularly.

24. I have the necessary resources (e.g., space, equipment) to practice yoga at the hostel/college.

25. I am willing to seek guidance from a yoga instructor or online resources to start my practice.

26. I feel prepared to handle any physical challenges that might arise from practicing yoga daily.

27. I believe that the benefits of yoga outweigh the effort required to practice it daily.

28. I feel confident in my ability to stay committed to a daily yoga routine over the long term.

Section D: Barriers Preventing Yoga Practice

29. Select all that apply

  (a) Time constraints

  (b) I feel unmotivated to wake up early

  (c) I feel yoga is too boring

  (d) I do not have enough energy (physical tiredness)

  (e) I don’t have a quiet and comfortable space

  (f) I cannot practice yoga without guidance

  (g) I find yoga physically challenging

  (h) I find it hard to stay consistent with yoga practice over time

  (i) I believe that yoga is not as effective as other forms of exercise

Section E: Perception on Integrating Yoga Into the MBBS Program

30. I believe that daily yoga sessions should be part of the MBBS curriculum.

31. I think that yoga sessions should include an educational component to explain the health benefits of each practice.

32. Participation in yoga sessions should be:

  ☐ Mandatory

  ☐ Optional

33. Participation in yoga sessions should be:

  (a) Early morning before academic classes

  (b) Between 8 am and 5 pm during academic sessions

  (c) In the evening, after academic classes

  (d) Flexible, allowing students to choose their preferred time slots

34. I believe that the duration of yoga sessions should be

  (a) 10-15 minutes

  (b) 20-30 minutes

  (c) 45-60 minutes

  (d) 60 minutes

35. I believe yoga classes should be conducted for (select all that apply):

  (a) Once a week

  (b) 2 days a week

  (c) 3 days a week

  (d) 5 days a week

  (e) Daily

36. Which format do you think would work best for yoga sessions? (select all that apply):

  (a) Online - Live sessions with a qualified instructor (Synchronous classes)

  (b) Online - Pre-recorded video sessions for flexible timing (Asynchronous classes)

  (c) Physical classes

37. I believe that yoga sessions should include (select all that apply):

  (a) Physical postures

  (b) Breathing exercises

  (c) Meditation

  (d) Focused more on stress relief

38. I believe that improvement in students' physical and mental health should be assessed periodically.

Please respond using a 5-point Likert scale: 1 = Strongly Disagree, 2 = Disagree, 3 = Neutral, 4 = Agree, 5 = Strongly Agree

Section F: Open-Ended Question

Write your view regarding yoga and its integration in daily life.

________________________________________________________________________________________________________________________________________________________________________________________________________________________
